# Polyagglutinability phenomenon: a case report and review of the literature

**DOI:** 10.1186/s13256-023-04072-z

**Published:** 2023-08-16

**Authors:** Noussaiba Azzi, Nabiha Trougouty, Rachid seddik

**Affiliations:** Laboratoire d’hématologie, CHU Mohammed VI, Faculté de Médecine et de Pharmacie d’Oujda, Université Mohammed Premier, Oujda, Morocco

**Keywords:** Polyagglutinability, Immunology, Hematology, Transfusion, Cryptic antigen

## Abstract

**Background:**

Polyagglutinability of red blood cells is a rare immunological phenomenon, it is due to a cryptic antigen that is abnormally present on the surface of red blood cells. The aim of our work is to shed light on polyagglutinability, which is still poorly understood cause of discordance between the cell and serum tests and can sometimes have harmful transfusion consequences.

**Case presentation:**

We report the case of a 70-year-old African patient admitted for management of hemolytic anemia.

**Results:**

During the erythrocyte grouping, a discordance between the cell and serum tests was observed, with polyagglutinability for the RH phenotype, a positive AB control, and even a positive control. The direct antiglobulin test and the Coombs test were also positive. The same results were obtained even after washing the red blood cells and incubating them at 37 °C for 30 min. For transfusion purposes, erythrocyte genotyping was performed, and the patient was transfused with an A+ red blood cell unit with an RH Kell-compatible phenotype.

**Conclusion:**

Polyagglutinability should always be taken into account when grouping anomalies are encountered. Although it may not show any symptoms, hemolysis is frequently observed during transfusions.

## Introduction

Polyagglutinability of erythrocytes is a rare immunological phenomenon. It is due to a cryptic antigen that is unmasked or abnormally presented on the surface of red blood cells. This antigen is recognized by an irregular natural antibody present in most human sera (otherwise compatible in the ABO system) [1].

For a long time, the term panagglutinability was wrongly used to describe polyagglutinability. In fact, in polyagglutinability, red blood cells are agglutinated by all normal human sera, while in panagglutinability, the red blood cells of a normal individual (the I gene) are agglutinated by a serum containing an IgM type antibody (called anti-I) that agglutinates at an optimal temperature between 15 and 20 °C. In 1927, Mr. Thomsen was the first to observe a polyagglutinated sample [2]. By studying the possible cause, he discovered that it was the action of a bacterial enzyme that modified the cell membrane structure of red blood cells and caused a T-type antigen to be unmasked. This antigen is therefore systematically recognized by anti-T antibodies present in the sera of almost all individuals. These phenomena are classified into two categories: hereditary and acquired, suspected in an infectious or malignant context, sometimes accompanied by anemia [2].

The diagnosis of these phenomena is based on the use of a panel of specific lectins. Polyagglutinability is one of the main causes of discordance between the globular and serological tests in immuno-hematological analysis, and its transfusional impact has been widely evaluated, leading to the recommendation to transfuse in plasma-depleted blood products, especially for T polyagglutinability [3].

## Case presentation

We report the case of a 70-year-old African patient admitted for management of hemolytic anemia. His medical history was marked by type 2 diabetes and two daughters with anemic syndrome.

The history of the disease dates back 2 years to the onset of generalized pruritus and mucocutaneous icteria; on examination, the patient presented hepatosplenomegaly and bilateral cervical adenopathy; the patient was hospitalized in the internal medicine department and underwent a whole-body computed tomography (CT) scan revealing abdominal adenopathy and splenomegaly; the evolution was marked by development of thrombophlebitis of the inferior member. Since then, the patient had been hospitalized several times for management of hemolytic anemia.

Laboratory tests showed a regenerative normochromic normocytic anemia with a hemoglobin level of 8.0 g/dL (reticulocytes 239,000), elevated LDH of 328 UI/L, and decreased haptoglobin. The rest of the evaluation was unremarkable.

**Fig. 1:**
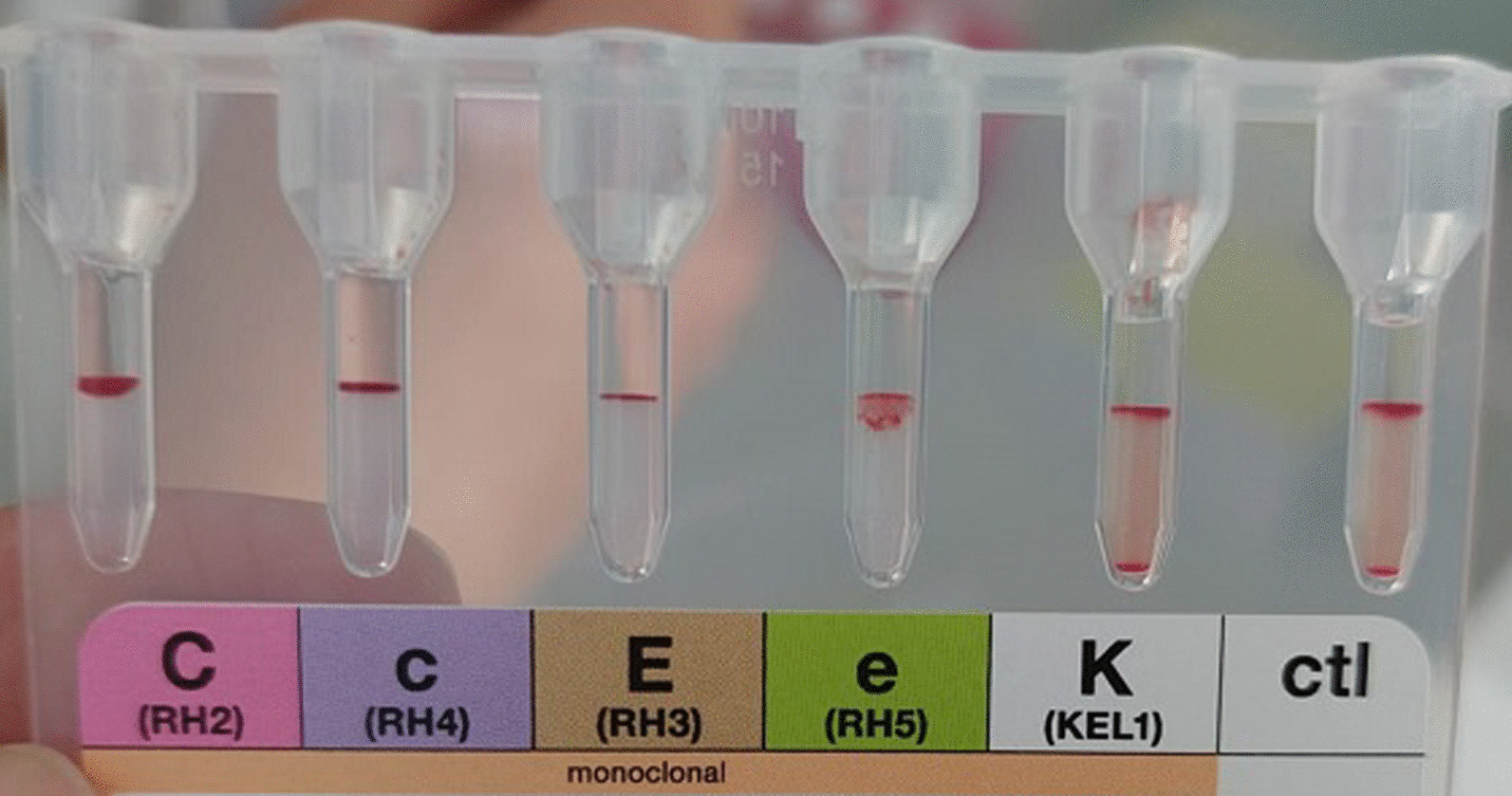
Rh Kell phenotype at admission

In red cell type testing by gel card filtration (BIO-RAD), a discordance between cellular and serum testing was observed: for cellular testing, antibodies anti-A (4+), anti-B (2+), and anti-AB (4 +) were detected, whereas for serum testing, Hema A was (1+) and Hema B was (3+) (Table 1). In addition, agglutination was detected with all Rh antibodies, even if the intensity of the agglutination varied (Fig. 1).

**Table 1:** Globular and serum test

	Globular test			Serum test		AB control
Groups	Anti A	Anti B	Anti AB	Cells A	Cells B	+
	4+	2+	4+	+	3+

**Fig. 2:**
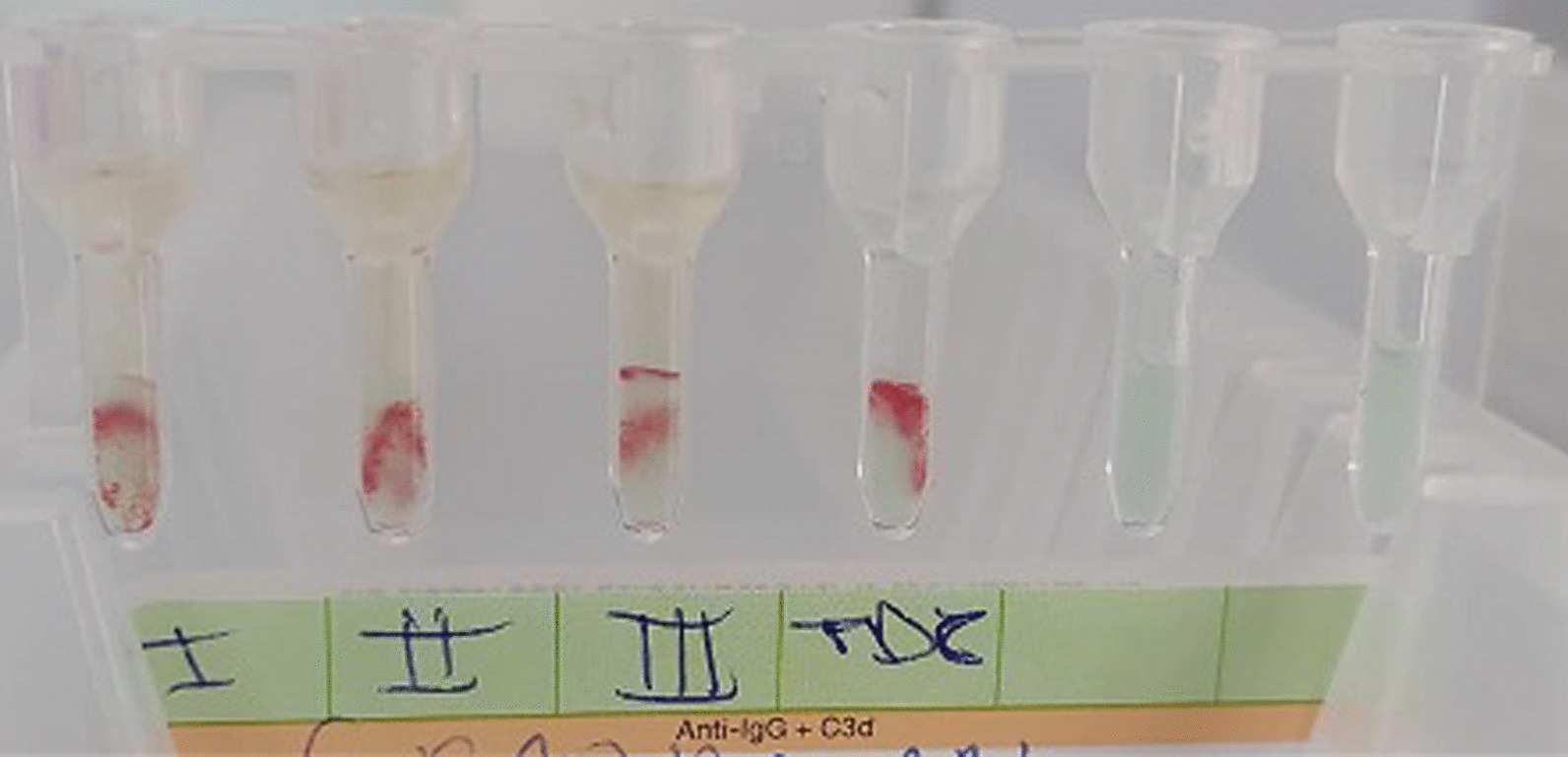
Positive irregular agglutinin test and Coombs Direct test

**Fig. 3:**
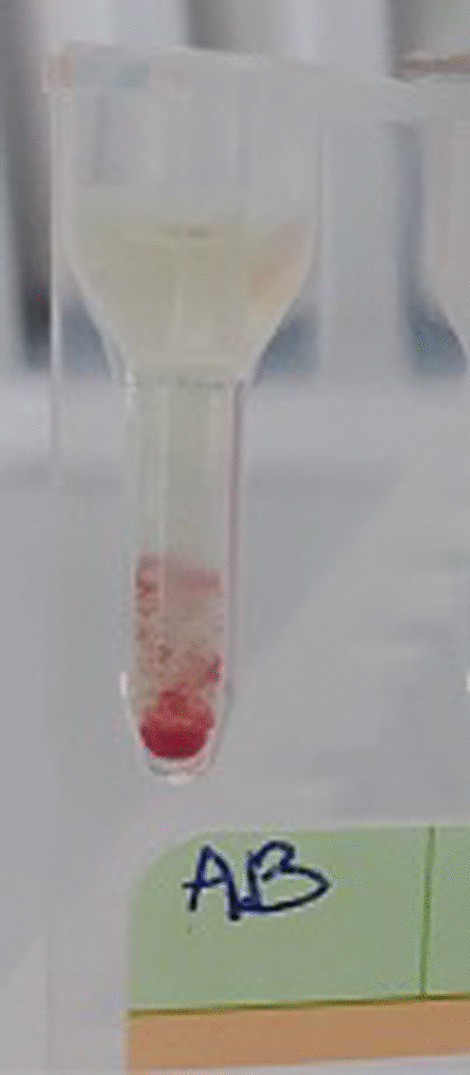
Positive AB control using gel card filtration

**Fig. 4:**
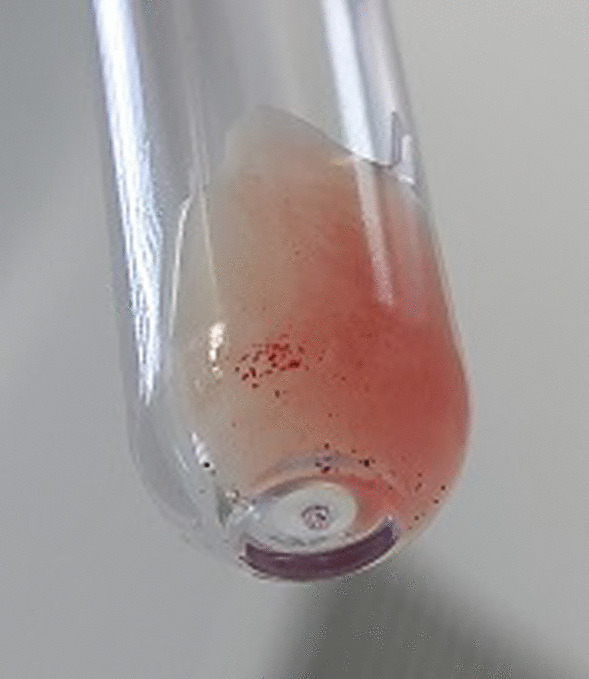
Positive AB control on tube

We obtained the same results even after a series of red cell washes and incubation at 37 °C for 30 min. An irregular antibody test (IAT) and direct Coombs test were performed and were positive (Fig. 2). The controls showed a positive AB control (Figs. 3 and 4).

**Fig. 5:**
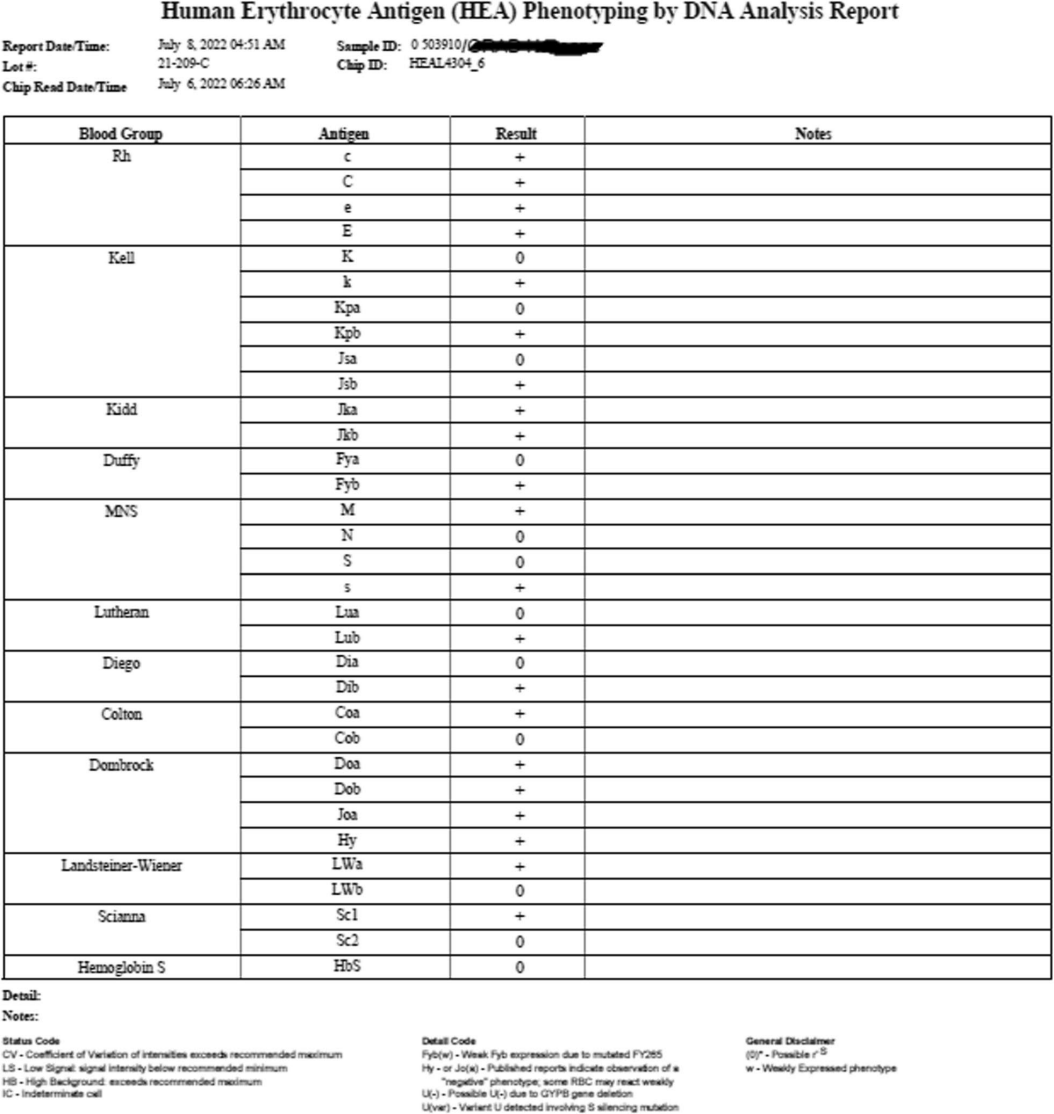
Erythrocyte genotyping

The patient received corticosteroid therapy (bolus and maintenance treatment), and a blood grouping was performed after 4 weeks with the same findings and a positive AB control. For transfusion needs, erythrocyte genotyping was performed (Fig. 5) to validate the Rh phenotype, and the patient was transfused with compatible ABO Rh Kell blood. The patient died after a stay in the intensive care unit with septic shock, therefore; other tests to explore and confirm polyagglutinability, such as adsorption tests and lectin tests, were not performed.

## Discussion

An erythrocyte is considered polyagglutinable when it agglutinates with most human AB sera that contain natural antibodies directed against different cryptic or transformed antigens implicated in these phenomena. The polyagglutinability phenomenon is now very rarely observed due to the widespread use of monoclonal reagents for blood grouping, which do not contain antibodies directed against the antigens responsible for polyagglutinability [4].

These phenomena are classified into two categories: hereditary polyagglutinabilities (such as HEMPAS, NOR, CAD) and acquired polyagglutinabilities (such as T, Tk, Th, Tx, Tn, acquired B, and Va), suspected in an infectious or malignant context.

### Hereditary polyagglutinabilities

#### HEMPAS type polyagglutinability: hereditary erythroblastic multinuclearity with a positive acidified serum test

**Fig. 6:**
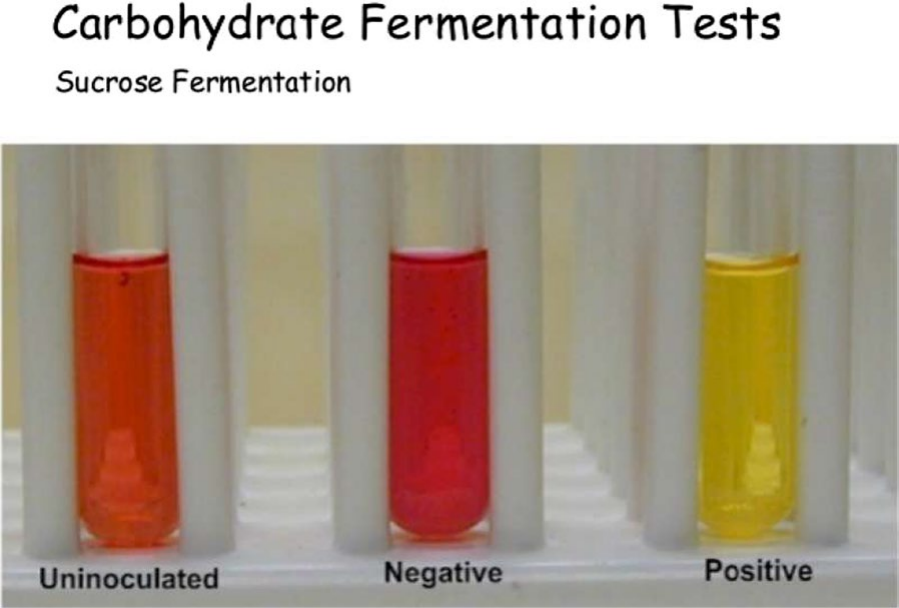
Sucrose test

This type of polyagglutinability is encountered in individuals with type II dyserythropoiesis. During this condition, nuclear anomalies (often multinucleated erythroblasts) and red blood cell membrane anomalies (abnormal glycosylation) have been described. The biochemical nature of the Hempas antigen is currently unknown. This antigen may be present without any clear blood anomalies, but it should be noted that in these cases the morphology of the erythroblasts has not been studied [5]. It should also be noted that these erythrocytes are sensitive to the direct action of complement. This abnormal complement sensitivity is linked to a membrane cholinesterase deficiency [5]. The sucrose test aims to highlight excessive sensitivity of erythrocyte membrane to complement lytic action. Patient erythrocytes are incubated with sucrose and serum (providing complement) from a normal ABO-compatible individual. HEMPAS+ subjects have a negative sucrose test (Fig. 6). Furthermore, none of the lectins currently used can detect the HEMPS antigen. HEMPAS+ subjects present a quantitative decrease in Ag H and a considerable increase in Ag i and I, resulting in significant hemolysis in the presence of Anti-I and Anti-i antibodies. They also exhibit an increase in agglutinability after treatment with papain [6].

#### Cad type polyagglutinability:

The Cad antigen was first described in 1968 by CAZAL and colleagues. These antigens are very rare, and their frequencies vary depending on the studied populations (0.03% in Japan); (0.08% in France); (0.24% in Thailand). Genetic transmission of the Cad antigen occurs in a dominant mode and independently of the ABO system and other systems. On the biochemical level, the immuno-dominant sugar of the Cad antigen is *N*-acetylgalactosamine, just like the A1, A2, Sda, and Tn antigens. The Cad antigen shows high similarity with the Sda antigen of normal red blood cells. However, Cad (+) subjects have a stronger amount of Sda antigen than Cad (−) subjects. The Cad antigen is recognized by its reactivity with the lectin Anti A1 and Anti Cad extracted from DOLICHOS BIFLORUS. Currently, three Cad antigen varieties are recognized: Cad 1, 2, and 3, defined in descending order of their polyagglutinability.

#### NOR type polyagglutinability

The first case of hereditary NOR polyagglutinability was reported in 1982. Using a panel of lectins, the authors identified a new type of polyagglutinability that was enhanced after treatment of NOR erythrocytes with proteases and inhibited by sheep hydatid cyst fluid or "avian P1" substance. A few years later, it was demonstrated that sialidase promotes this type of polyagglutinability and that treatment of the red blood cells with galactosidase suppresses it [7].

NOR erythrocytes possess unique neutral glycosphingolipids, and high-performance thin-layer chromatography (HPTLC) of these glycosphingolipids showed the presence of two bands strongly colored by isolectin IB4 isolated from *Griffonia simplicifolia* (GSL-IB4).

NOR+ subjects do not react with the snail and seed lectins used to identify other subtypes of polyagglutinability [8].

#### Hemoglobin M-Hyde Park

Hemoglobin M-Hyde Park is a rare type of hereditary hemoglobinopathy that predisposes the patient to methemoglobinemia and hemolytic anemia. The erythrocytes of patients with this disorder agglutinate when they come into contact with most adult sera and are therefore polyagglutinable. Hemoglobin M is the only known variant of hemoglobin associated with polyagglutinability.

The erythrocytes of these patients have aberrant synthesis of certain parts of the band 3 complex, exposing them to antibody attack and elimination by agglutination. These red blood cells also show strong agglutination with G Iycine soju and *Sophoru juponicu*, are weakly reactive with *Sulviu horminum* and Bundeirueu sitnplifolicu II, and show no reaction with Dolichos btf [9].

#### Tr type polyagglutinability

Described by Reid *et al*. in 1998, this was a 15-year-old boy with mild hemolytic anemia, Bernard-Soulier syndrome, and intermittent neutropenia. The authors concluded that the patient's red blood cells had a glycosylation disorder, particularly a reduction in *N*-acetylneuraminic acid, exposing an antigen with a galactose residue, probably the result of altered glycosyltransferases. This type of antigen has a strong affinity for *Gr. Simplicifolia* [10].

### Acquired polyagglutinabilities

#### Type T polyagglutinability

The T antigen is a normal antigenic element of erythrocytes that is usually masked by other structures; it is a cryptic antigen. The corresponding antibody, anti-T, is present in all normal human sera (Immuno Dominant sugar: beta-Galactose). The T type, the earliest recognized, is due to the action of bacterial neuraminidase (e.g., Vibrio cholerae and Clostridium perfringens) on the erythrocyte membrane, the enzyme cuts the glycoprotein and glycolipid chains of the erythrocyte membrane to expose the T antigen [11].

The diagnosis of type T polyagglutinability is easy since specific anti-T activity was recognized in lectin extracted from Arachis Hypogaea and Glycine Soja. A human reagent can be obtained by fixing and eluting an AB serum on OT erythrocytes.

Unmasking the T antigen can pose a grouping difficulty when using polyvalent human test sera not cleared of their anti-T activity; there is often a discrepancy between the Beth-Vincent and Simonin tests. The use of monoclonal antibodies can overcome this difficulty. These phenomena are often accompanied by clinical and laboratory evidence of hemolysis. Thus, a positive direct Coombs test is frequently observed, and elution allows the isolation of an anti-T antibody [12].

In a study involving 10,000 patients, 0.01% of severe infections were associated with type T polyagglutinability. This type of polyagglutinability has also been described in CLL and MM.

#### Type Tk polyagglutinability

This is an infectious polyagglutinability first described in 1972 by BIRD in a woman with a urinary tract infection caused by *E. coli*. It is an acquired, transient polyagglutinability that disappears with the resolution of the infectious episode. Serologically, the characteristics of the Tk transformation are identical to those of the T transformation except that Tk red blood cells are agglutinated by polybrene and not by T. It appears that Tk is a minor form of the T transformation [6]. Polyagglutinability phenomena of type Tk and Th have been found in patients with solid tumors with or without sepsis. Acquired type B PA: Acquired type B is characterized by the appearance, in a subject with A1 blood type, most often in the context of digestive infection associated with colonic cancer, of reactivity with anti-B sera. The responsible organism produces a deacetylase that transforms the immunodominant sugar of the A antigen (*N*-acetylgalactosamine) into the immunodominant sugar of the B antigen (galactosamine, forming pseudo-B from A) [13]. In this type of polyagglutinability, the fixation-elution test of anti-B is positive, and reactions with anti-T, anti-Tn, anti-Cad, and AB sera are negative. It should be noted that polyagglutinabilities associated with a malignant process are related to the absence of synthesis of the sugar chain of glycophorins, leaving a normally cryptic antigen exposed.

#### Acquired B polyagglutinability

Type B acquired is characterized by the appearance, in a subject of group A1, most often in the context of digestive infection associated with colonic cancer, of reactivity with anti-B sera. The responsible germ produces a deacetylase that transforms the immunodominant sugar of Ag A (*N*-acetylgalactosamine) into galactosamine, the immunodominant sugar of Ag B (pseudo B forming at the expense of A) [13]. In this type of polyagglutinability, the fixation-elution test of an anti-B is positive, and the reactions with anti-T, anti-Tn, anti-Cad, and AB serum are negative. It should be noted that polyagglutinabilities associated with a malignant process are linked to the absence of synthesis of the sugar chain of glycophorins, leaving a normally cryptic Ag exposed.

#### Tx polyagglutinability

The Tx cryptantigen was first described in a patient admitted with hemolytic anemia and recurrent pneumococcal infection. The detection of this type of PA requires a panel of lectins including at least Vicia cretica and/or Medicago disciformis. Transfusion of a deplasmatised RBC appears to improve hemolysis in some patients, highlighting the importance of screening and identifying these antigens [14].

#### Th polyagglutinability

It is a form of weak activation of the T antigen, first described in 1978. The Th antigen results from the action of bacterial neuraminidase; the most involved germs are E. coli, Clostridium, and Proteus. A fatal case of severe intravascular hemolysis occurred in a patient with peritonitis due to a perforated colonic tumor. Th activity is significantly reduced after treatment of RBCs with papain, it is demonstrated by the *Arachis hypogaea* lectin and does not react to Glycine soja or *Griffonia simplicifolia* [6].

#### VA polyagglutinability

In 1977, a new form of polyagglutinability called VA (Vienna) was discovered in a 20-year-old man with hemolytic anemia. The patient's RBCs were not agglutinated by *Arachis hypogaea*, which distinguished them from other types of PA induced by microbial agents (T, Th, Tk, and TX). VA erythrocytes aggregated normally in polybrene solutions, indicating a normal concentration of lactic acid. The MN antigens were fully expressed; however, H receptors were decreased [15]. Cases of Tk type PA associated with VA PA have been reported. In this case, the reduction in erythrocyte H activity could be attributed to the action of bacterial alpha-fucosidase.

#### Specificity of the Tn antigen

The Tn polyagglutinability is distinguished from other acquired polyagglutinabilities by its persistent character and its frequent association with hematologic disorders such as hemolytic anemia, thrombocytopenia, and leukopenia. Furthermore, it has never been demonstrated that Tn polyagglutinability is linked to an infectious episode, and only this type of polyagglutinability has been described in patients with acute myeloid leukemia [16].

In the majority of cases, this polyagglutinability presents a characteristic double population aspect, and it is possible to separate Tn from non-transformed erythrocytes. This is due to the absence of T enzyme leading to a lack of conversion of the Tn substrate, resulting in a pseudo A double population image [16].

These various characteristics suggest that Tn reactivity could be due to a somatic mutation occurring in certain bone marrow stem cells. Anti-Tn activity exists in all normal human serums and is also present in the lectin extracted from *Dolichos biflorus*, regardless of the ABO group.

Treatment with papain suppresses Tn reactivity. Tn erythrocytes have a reduced sialic acid content but remain serologically distinct from other sialic acid-deficient types.

It is estimated that our patient had acquired polyagglutinability, given the improvement in grouping after the infectious episode, and the clarity of the globular test, allowing for a blood group A determination. However, the determination of the exact phenotype of PA should be obtained through specific lectins.

## Conclusion

Polyagglutinability is a rare phenomenon in transfusion practice. Although it may be asymptomatic, hemolysis is often observed during transfusion. Exceptionally, complications such as DIC or hemolytic uremic syndrome have been described [17]. When grouping aberrations are identified, polyagglutinability should always be considered. The discovery of cryptic antigens may reveal an infectious or tumoral pathology. Transfusion of RBCs non reduced in plasma seems to aggravate the hemolysis phenomenon in some of the patients, which underlines the importance of testing and identifying these antigens.

## Data Availability

Not applicable.

## Abbreviations

RBCs: Red blood cells
RH Kell: Rhesus Kell
IgM: Immunoglobuline type M
LDH: Lactate dehydrogenase
IAT: Irregular antibody test
PA: Polyagglutinability
DIC: Disseminated intravascular coagulation
